# Whole-Genome Sequencing and Genomic Characterization of a Multi-Drug Resistant Phenotype of *Listeria monocytogenes* Isolated from Pet Food

**DOI:** 10.3390/microorganisms14051097

**Published:** 2026-05-12

**Authors:** Antonia Mataragka, Marios Mataragas, Nikolaos Tzimotoudis, Ioannis Galiatsatos, Panagiota Stathopoulou, Spiros Paramithiotis, John Ikonomopoulos, Nikolaos D. Andritsos

**Affiliations:** 1 Department of Food Science and Technology, School of Agricultural Sciences, University of Patras, 2 G. Seferi Str., GR-30100 Agrinio, Greece; antonia.mataragka@gmail.com; 2 Laboratory of Anatomy and Physiology of Farm Animals, Department of Animal Science, School of Animal Biosciences, Agricultural University of Athens, 75 Iera Odos Str., GR-11855 Athens, Greece; ikonomop@aua.gr; 3 Department of Dairy Research, Institute of Technology of Agricultural Products, Hellenic Agricultural Organization “DIMITRA”, 3 Ethnikis Antistaseos Str., Katsikas, GR-45221 Ioannina, Greece; 4 Hellenic Army Biological Research Centre, 6 Taxiarchou Velliou Str., P. Penteli, GR-15236 Attica, Greece; n.p.tzimotoudis@army.gr; 5 Laboratory of Systems Microbiology and Applied Genomics, Department of Sustainable Agriculture, School of Agricultural Sciences, University of Patras, 2 G. Seferi Str., GR-30100 Agrinio, Greece; jgalia96@gmail.com (I.G.); panstath@upatras.gr (P.S.); 6 Department of Biological Applications and Technology, School of Health Sciences, University of Ioannina, Ioannina Campus, GR-45110 Ioannina, Greece; paramithiotis@uoi.gr

**Keywords:** food safety, genomic analysis, *Listeria monocytogenes*, multi-drug resistance, pet food, virulence, whole-genome sequencing

## Abstract

*Listeria monocytogenes* is already a well-known foodborne bacterial pathogen, ubiquitously dispersed not only in the food production environment but also in the primary animal production environment as well. The present study performed whole-genome characterization of the multidrug-resistant (MDR) *L. monocytogenes* strain BF11, previously isolated from raw pet food and phenotypically described for antimicrobial resistance. To this end, the genomic analysis performed on the isolate confirmed the pathogen’s designation as a serotype 1/2b strain belonging to ST5 and CC5 (Lineage I), carrying multiple MDR genes, stress-related genes, and mobile genetic elements, despite the absence of plasmids. The strain is phylogenetically closely related to Lineage I epidemic strains (e.g., F2365), as it has a full-length *inlA* and a functional *prfA*, rendering it capable of invading human cells and marking its high virulence. Overall, this strain may represent a potentially novel genomic profile when core genome multilocus sequence typing (cgMLST) is used, although further data from additional isolates would be required to confirm its classification within a new Complex Type, while displaying a hybrid unique profile. It is an evolved ST5 *L. monocytogenes* strain that has acquired genetic material conferring a “clinical signature” (Lineage I-like) and an extensive resistance network. Therefore, presence of *L. monocytogenes* strain BF11 in pet food is alarming, since such hybrid strains often evade surveillance monitoring as they do not fit strictly into classical categories, posing a serious food safety and public health threat in the concept of One Health.

## 1. Introduction

*Listeria monocytogenes* is a major Gram-positive foodborne pathogen responsible for listeriosis, a severe illness posing significant public health risks, particularly to pregnant women, neonates, the elderly, and immunocompromised individuals [1,2,3]. This bacterium is widely distributed in nature and occurs in both food production facilities and primary animal production systems [3,4]. Its capacity to survive and proliferate under adverse conditions, including refrigeration temperatures, elevated salt concentrations, and acidic environments, contributes to its persistence in diverse ecological niches [5,6].

The clinical management of listeriosis relies on early administration of antibiotics, with ampicillin or penicillin often combined with gentamicin as first-line therapy, and trimethoprim–sulfamethoxazole as an alternative for penicillin-allergic patients [7,8]. Nevertheless, antimicrobial resistance (AMR) in *L. monocytogenes* has become a growing concern in both clinical and food safety contexts [9]. While intrinsic resistance to certain antimicrobial classes is well established, acquired resistance determinants have also been reported and may contribute to multidrug-resistant (MDR) phenotypes [10,11]. Resistance to clinically relevant antibiotics, including tetracycline, erythromycin, clindamycin, fluoroquinolones such as ciprofloxacin, and sulfonamides, has been detected among isolates from food, environmental, and clinical origins [12,13,14]. In some cases, these phenotypes are mediated by acquired genes such as *tet*(*M*), *erm*(*B*), and *lnu*(*B*), frequently associated with mobile genetic elements [15,16,17].

The pathogenicity of *L. monocytogenes* is mediated by a diverse set of genes, many of which are organized within pathogenicity islands such as Listeria Pathogenicity Island 1 (LIPI-1). Key factors include the transcriptional regulator *prfA*, *hly*, *inlA*, *inlB*, *plcA*, and *actA* [18,19,20]. Expression of these factors enables host–cell invasion, intracellular survival, and dissemination within host tissues [21]. Moreover, the ability of *L. monocytogenes* to form biofilms on abiotic surfaces common in food-processing environments contributes to its persistence and recurrent contamination. Biofilm-associated cells may show increased tolerance to disinfectants and sanitizers, complicating eradication efforts and contributing to stress adaptation [22,23,24,25].

Most genomic and AMR studies of *L. monocytogenes* have focused on foods intended for human consumption, food-processing environments, clinical isolates, livestock-associated matrices, and environmental waters [26,27,28,29,30,31,32,33,34]. In comparison, pet food remains less extensively characterized, despite its relevance at the food–feed–household interface. Unlike many foods intended for human consumption, raw pet foods may be handled repeatedly in domestic settings, stored alongside household items, and dispensed through bowls or utensils that can become points of cross-contamination. This is particularly relevant for households or occupational settings involving vulnerable individuals, veterinary personnel, animal handlers, or service dogs [35,36].

In a previous study, *L. monocytogenes* strain BF11 was isolated from biologically appropriate raw food (BARF) intended for military service dogs and exhibited an MDR phenotype, with resistance to ciprofloxacin, meropenem, penicillin, trimethoprim–sulfamethoxazole, and tetracycline [37]. The present study aimed to perform whole-genome characterization of this MDR raw pet-food isolate and to evaluate whether its resistance phenotype co-occurs with genetic features that increase its relevance for One Health surveillance, including association with clinically relevant lineage background, virulence potential, stress adaptation, mobile genetic elements, and persistence-associated traits. Whole-genome sequencing was therefore used to determine the serogroup, multilocus sequence typing (MLST) and/or core genome MLST (cgMLST) profile, resistance-associated loci, virulence-associated genes, prophage content, putative CRISPR-Cas elements, plasmid status, and comparative genomic position of BF11, thereby enabling genomic risk characterization of this MDR *L. monocytogenes* strain from raw pet food.

## 2. Materials and Methods

### 2.1. Sampling Source and Microbiological Analysis for Pathogen’s Isolation and Confirmation

A commercially available frozen raw meat-based pet food sample, marketed as a complete BARF diet for dogs, was collected by the Hellenic Army Biological Research Centre on 26 May 2022. According to the product label, it was intended for dogs of all breeds and ages and consisted mainly of poultry-derived ingredients, together with vegetables and added nutritional components. The product was supplied as a sealed 1 kg frozen retail package. The sample was analyzed in accordance with current microbiological criteria for the presence of *L. monocytogenes* among other microorganisms, as described elsewhere [37]. The detection and isolation of *L. monocytogenes* were performed according to the ISO 11290-1 protocol [38]. Briefly, the sample was primary enriched in half-Fraser broth (BioKar Diagnostics, Pantin, France) at 30 °C for 24 h, followed by secondary enrichment in full-Fraser broth (BioKar Diagnostics) at 37 °C for 48 h. The enriched culture was streaked in duplicate onto COMPASS^®^ Listeria agar (Biokar Diagnostics), an ALOA-type medium. Presumptive *L. monocytogenes* colony was confirmed through biochemical testing, including catalase reaction, oxidase test, motility test at 25 °C, rhamnose and xylose utilization, haemolysis reaction, and CAMP test.

### 2.2. Antimicrobial Susceptibility Testing

The antimicrobial susceptibility profile of the confirmed *L. monocytogenes* isolate was determined using the Kirby–Bauer disk diffusion method [39] on Mueller–Hinton agar plates supplemented with 5% defibrinated horse blood and 20 mg/L β-NAD (MH-F; Bioprepare, Keratea, Attica, Greece), in accordance with the guidelines of the European Committee on Antimicrobial Susceptibility Testing (EUCAST v.15.0) [40]. The minimum inhibitory concentration (MIC) for antimicrobials to which the isolate showed resistance was further determined using the E-test method (BioMérieux, Marcy-l’Étoile, France) [41]. The antimicrobial panel included ampicillin (AMP, 2 μg), benzylpenicillin (P, 1U), ciprofloxacin (CIP, 5 μg), erythromycin (E, 15 μg), meropenem (MEM, 10 μg), trimethoprim-sulfamethoxazole (SXT, 25 μg), and tetracycline (TE, 30 μg) (Oxoid, Basingstoke, UK).

### 2.3. Molecular Serotyping

Genomic DNA was extracted from the bacterial isolate using a commercial kit (Nucleospin^®^ Tissue, Macheray-Nagel GmbH & Co. KG, Düren, Germany) according to the manufacturer’s instructions. The major serogroup (IIb) was determined by multiplex PCR which targets the *L. monocytogenes* genes *prs*, *lmo0737*, *lmo1118*, *ORF2819*, and *ORF2110* to differentiate between the major serotypes 1/2a, 1/2b, 1/2c, and 4b [42].

### 2.4. Bioinformatic Analysis

Assembly and annotation of the genome were carried out on the Bacterial and Viral Bioinformatics Resource Center (BV-BRC) platform [43] with the Genome Assembly Service, and the proposed pipeline, i.e., Flye as long-read assembler [44] and correction (polishing) of the assembly errors with minimap2 [45] and Racon [46], and the Genome Annotation Service, respectively. Species confirmation was performed using the Type (Strain) Genome Server (TYGS) (https://tygs.dsmz.de/, accessed on 16 December 2025) [47], the Average Nucleotide Identity (ANI) as estimated by the OrthANI software (https://www.ezbiocloud.net/, accessed on 16 December 2025) [48], and the BIGSdb/rMLST tools [49,50]. Assembly quality was evaluated with QUAST [51]. Other quality checks performed involved Kraken 2 contaminants [52], total assembly length deviation, percentage of typing loci detected, percentage of complete BUSCO genes [53], CheckM [54,55] and ContEst 16S [56] for completeness and contamination of the assembled genome. Pathogenicity, virulence and antimicrobial resistance of the strain was analyzed with the following bioinformatic tools: PathogenFinder [57], AMRFinder [58], ResFinder [59,60], VirulenceFinder along with the reference database VFDB [61]. The identification of mobile genetic elements and plasmids were done with MobileElementFinder [62] and PlasmidFinder [63], respectively. MLST [64] was employed for sequence type (ST) classification. Finally, CRISPR-Cas and prophages were searched through the CRISPRCasFinder [65] and PHASTEST [66] webtools, respectively. All the aforementioned tools and software were run through the use of the Center for Genomic Epidemiology (CGE) services (http://www.genomicepidemiology.org/services/, accessed on 16 December 2025), the European public Galaxy server (https://usegalaxy.eu/, accessed on 16 December 2025) [67], and the Bionumerics software v.8.1. (bioMérieux, Marcy l’ Etoile, France).

All bioinformatic tools and platforms were used with their latest available stable versions at the time of analysis (December 2025), including Flye (v2.9), minimap2 (v2.24), Racon (v1.5.0), QUAST (v5.2.0), Kraken 2 (v2.1.3), BUSCO (v5.4.7), CheckM (v1.2.2), AMRFinder (v3.11.2), ResFinder (v4.1), VirulenceFinder (v2.0), MobileElementFinder (v1.0.3), PlasmidFinder (v2.1), CRISPRCasFinder (v4.2.20), and PHASTEST (accessed December 2025). Analyses performed through web-based platforms (e.g., BV-BRC, CGE, and Galaxy) utilized the versions available on the respective servers at the time of access. Reference genomes used for phylogenomic and comparative analyses were retrieved from the NCBI and BV-BRC databases, and their accession numbers and associated metadata are provided in Supplementary Table S1.

## 3. Results

### 3.1. Bacterial Isolation and Confirmation

Phenotypic confirmation showed that *L. monocytogenes* strain BF11 was catalase-positive and oxidase-negative, exhibited characteristic tumbling motility at 25 °C, and produced β-hemolysis on sheep blood agar. The isolate showed a positive CAMP reaction with *Staphylococcus aureus* and a negative reaction with *Rhodococcus equi*. Carbohydrate utilization testing demonstrated L-rhamnose fermentation and absence of D-xylose utilization.

### 3.2. Antimicrobial Resistance Profile

*L. monocytogenes* strain BF11 exhibited an MDR phenotype, showing resistance to five antimicrobials; ciprofloxacin (CIP), meropenem (MEM), penicillin (P), trimethoprim–sulfamethoxazole (SXT), and tetracycline (TE), representing more than three antimicrobial classes. MIC testing confirmed resistance to MEM, P, SXT, and TE, with MIC values of 4 µg/mL, 4 µg/mL, >1024 µg/mL, and 48 µg/mL, respectively.

### 3.3. Serotyping of the Isolate

Serological typing classified strain BF11 as *L. monocytogenes* serogroup IIb strain, corresponding to serotypes 1/2b, 3b. The molecular and phenotypic serotyping results were fully concordant and consistently identified BF11 as belonging to serogroup IIb.

### 3.4. Genome Assembly and Quality Assessment

The genome of *L. monocytogenes* strain BF11 was sequenced using long-read technology and assembled into a single contig. The assembled genome had a total length of 2,993,949 bp and a G + C content of 37.98%. Assembly quality metrics indicated a high-quality genome suitable for downstream comparative and functional analyses. Genome completeness was estimated at 99.45%, with no detectable contamination or heterogeneity, and no ambiguous bases were identified (Table 1).

Advanced quality assessment confirmed the absence of taxonomic contamination, with 99.89% of typing loci detected and 99.20% of Benchmarking Universal Single-Copy Orthologs (BUSCO) genes identified as complete (Table 2).

The assembled genome contained 2948 predicted protein-coding sequences (CDSs), 68 tRNA genes, and 18 rRNA genes (Table 3).

**Figure 1:**
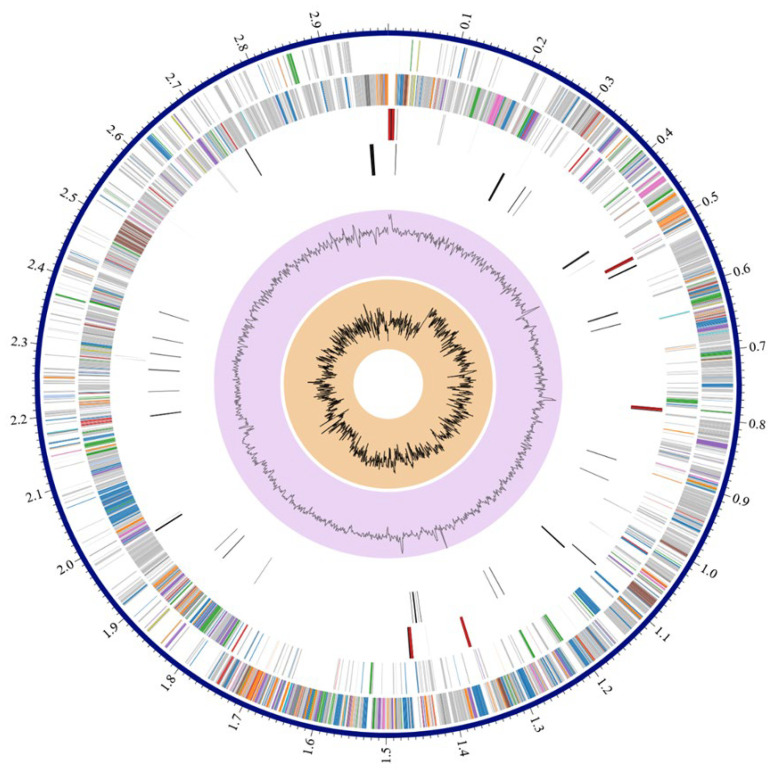
Annotation of the sequenced genome for *L. monocytogenes* strain BF11. From outer to inner rings, the contigs, CDS on the forward strand, CDS on the reverse strand, RNA genes, CDS with homology to known antimicrobial resistance genes, CDS with homology to known virulence factors, GC content and GC skew. The colors of the CDS on the forward and reverse strand indicate the subsystem that these genes belong to (see Figure 2).

**Figure 2:**
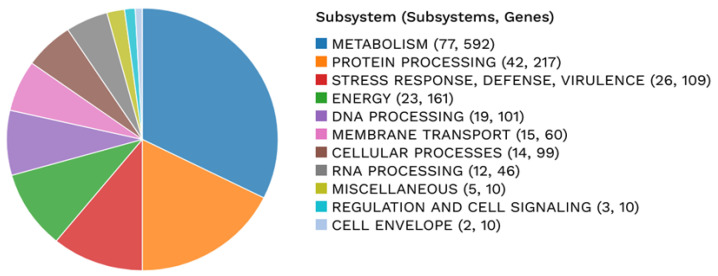
Annotation of the genome for *L. monocytogenes* strain BF11 including an analysis of the subsystems unique to the genome.

Among the annotated CDSs, 2423 were assigned a predicted function, while 525 were classified as hypothetical proteins. Functional annotation identified 784 proteins associated with Enzyme Commission (EC) numbers, 637 with Gene Ontology (GO) terms, and 551 mapped to Kyoto Encyclopedia of Genes and Genomes (KEGG) pathways. Pathosystems Resource Integration Center (PATRIC) annotation indicated that 2896 proteins belonged to genus-specific protein families (PLFams), and 2918 proteins were assigned to cross-genus protein families (PGFams). A circular graphical representation of the genome annotation, illustrating the distribution of coding sequences, RNA genes, and functional categories across the chromosome, is shown in Figure 1. In addition, an overview of functional subsystems, defined as sets of proteins collectively implementing specific biological processes or structural complexes, is provided in Figure 2.

### 3.5. Taxonomic Identification, MLST, and cgMLST Analysis

Taxonomic assignment using rMLST classified BF11 as a *L. monocytogenes* strain. Serotyping identified the strain as belonging to serogroup IIb, serovar 1/2b. MLST analysis classified BF11 as a sequence type ST5, clonal complex CC5 strain (Table 4). The strain was predicted to be a human pathogen with a probability of 90.9%.

cgMLST analysis was performed using a scheme of 1748 loci. No genomes within the PubMLST and BIGSdb-Pasteur databases were identified within a threshold of ≤10 allelic differences from BF11. Therefore, the strain did not cluster with any existing genomes within the applied threshold (≤10 allelic differences), suggesting that it may represent a potentially distinct cgMLST cluster within the currently available datasets. Analysis further revealed potentially novel alleles in 15 loci of the cgMLST scheme, indicating genetic divergence relative to previously deposited ST5 genomes.

### 3.6. Antimicrobial Resistance Phenotype and Genotypic Determinants

As previously mentioned, original phenotypic analyses demonstrated that *L. monocytogenes* strain BF11 exhibited resistance to CIP, MEM, P, SXT, and TE during antimicrobial screening, with MIC testing confirming resistance to MEM, P, SXT, and TE [37]. These results established the MDR phenotype of the strain. In silico analysis of antimicrobial resistance determinants identified a broad repertoire of resistance-associated genes and regulatory systems (Table 5).

Genes encoding antimicrobial inactivation enzymes, antimicrobial target modification, target replacement proteins, and regulatory systems were detected. The identified genes include both intrinsic (housekeeping-related) targets commonly associated with antimicrobial susceptibility (e.g., *alr*, *ddl*, *murA*, *gyrA*, *gyrB*) and acquired or resistance-associated determinants (e.g., *vga*(*G*), *norB*, *sul*, regulatory systems such as *liaFSR*). The latter are more directly linked to the observed MDR phenotype. No plasmids were detected in the genome, indicating that the resistance determinants identified were chromosomally encoded.

Genes associated with environmental stress tolerance were also identified, including the *bcrABC* cassette and Stress Survival Islet 1 (SSI-1). These loci are known to be involved in tolerance to quaternary ammonium compounds, acidic conditions, and high salt concentrations.

### 3.7. Virulence-Associated Genes and Pathogenicity Determinants

Analysis of virulence-associated loci revealed the presence of a complete LIPI-1, including *hly*, *plcA*, *plcB*, *actA*, and *prfA*. Multiple internalin genes were also detected, including *inlA*, *inlB*, *inlC*, *inlF*, *inlK*, and *inlP*.

Sequence analysis of the *inlA* gene demonstrated full-length integrity without premature stop codon mutations. BLAST analysis (https://blast.ncbi.nlm.nih.gov/Blast.cgi, accessed on 16 December 2025) showed 100% query coverage against the reference strain EGD-e, with 96% sequence identity and an E-value of 1.23 × 10^−298^, indicating a statistically significant and complete alignment. Sequence analysis also confirmed the presence of a functional *prfA* gene.

Analysis of virulence-associated databases further confirmed the presence of a complete virulence gene repertoire. In addition, sequence variation in *inlB* was identified compared to reference genomes, indicating the presence of potentially distinct allelic variants.

### 3.8. Mobile Genetic Elements, Prophages, and CRISPR-Cas System

Mobilome analysis identified multiple genes associated with recombination and genetic mobility, including *recT_2* and *int-Tn_2*. Several resistance- and virulence-associated genes showed 100% sequence identity to known reference genes.

Four integrated prophages were detected within the BF11 genome, occupying large genomic regions. Identified prophages belonged to the *Siphoviridae* family and included *Listeria* phages phi A006, phi LP-101, vB_LmoS_188, and B025. These prophage regions encompassed substantial portions of the chromosome.

A CRISPR-Cas system was also identified, containing three spacers. The presence of both multiple prophages and a CRISPR system indicates the coexistence of phage integration events and phage defense mechanisms within the BF11 genome.

### 3.9. Phylogenomic and Comparative Genomic Analyses

**Figure 3:**
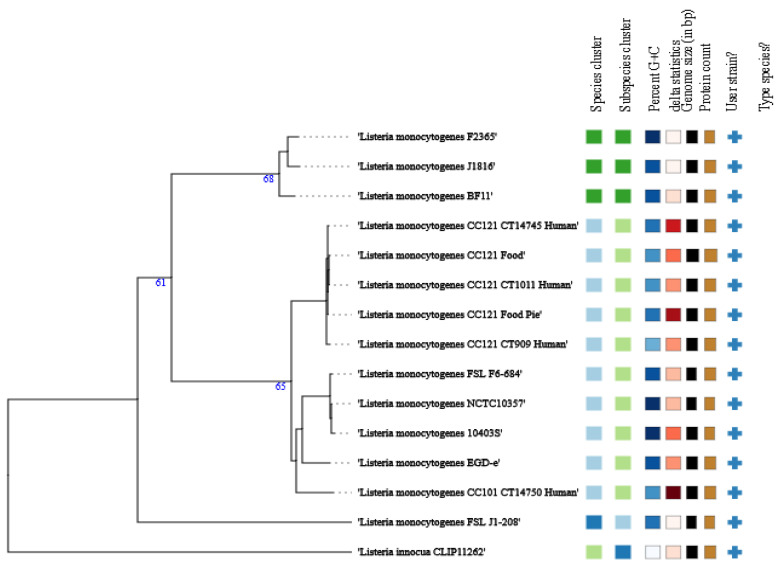
TYGS-generated WGS-based phylogram of the BF11 strain, as compared with other *Listeria* spp. reference strains. Strains with the same color are grouped together. All strains were added by the user (+) for comparison and were not TYGS-generated.

Whole-genome-based taxonomic analysis using TYGS assigned BF11 to *L. monocytogenes* (Figure 3).

**Figure 4:**
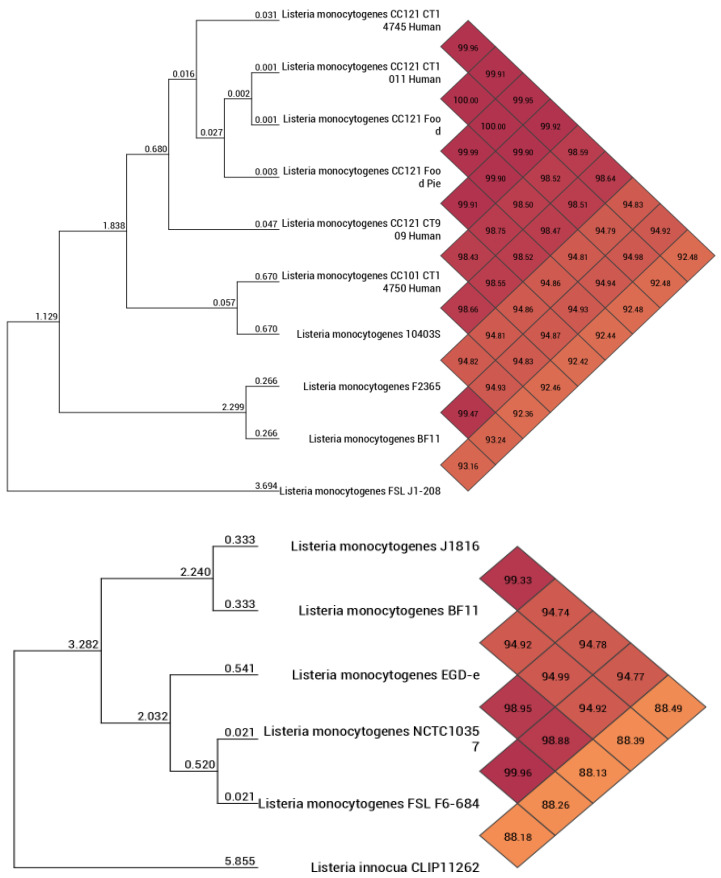
Heatmap–dendrogram graph (two parts, up and down) displaying the ANI values between the BF11 strain and different *Listeria* spp. strains. Strains with the same color are grouped together.

ANI analysis confirmed species-level identity, with ANI values exceeding the 95–96% threshold for species delineation when compared with reference *L. monocytogenes* genomes (Figure 4).

**Figure 5:**
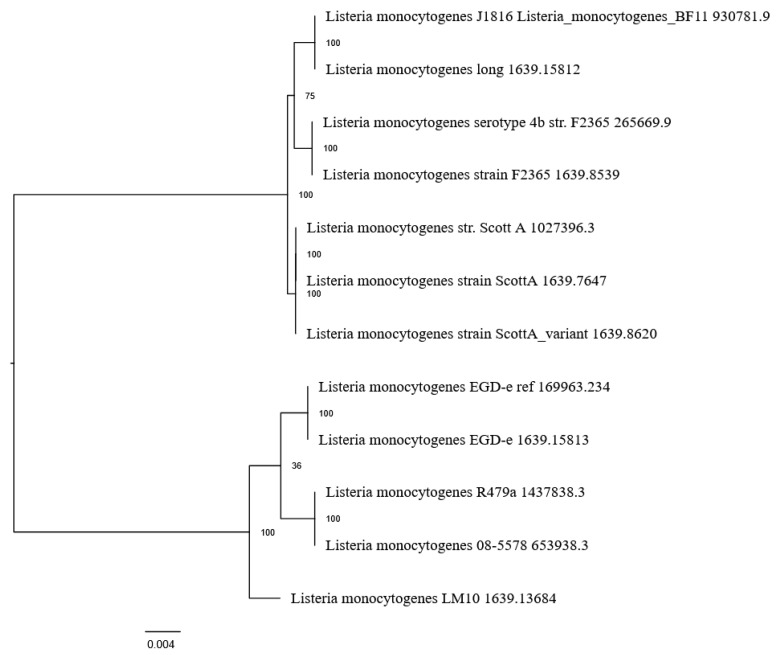
Phylogenetic dendrogram between the BF11 strain (the first two IDs 930781.9 and 1639.15812 at the beginning of the dendrogram) and selected reference *L. monocytogenes* strains. Multiple inputs per strain are displayed in the graph because BV-BRC platform contained the same strain with different ID. All IDs were included in the analysis to avoid missing data.

Proteome-based phylogenetic analysis revealed multiple clusters within the species, indicating proteomic divergence among strains (Figure 5). BF11 clustered in close proximity to the Lineage I reference strains F2365 and Scott A.

Comparative genome alignment analysis between BF11, a reference ST5 strain (08-5578), and the Lineage I strain F2365 identified multiple contiguous genomic regions shared between BF11 and F2365 that were absent from the reference ST5 genome, supporting the mosaic genome structure of BF11. Additional regions showed alignment between BF11 and the ST5 reference, indicating the presence of an ST5 genomic backbone with integrated genomic segments shared with Lineage I strains. Resistance-associated genes such as *vga*(*G*), *norB*, and *sul* were located within regions shared with the Lineage I reference strain.

## 4. Discussion

This study was structured as a whole-genome risk characterization of an MDR *L. monocytogenes* strain isolated from raw pet food. The main objective was to determine whether its MDR phenotype co-occurs with genomic features relevant to public health and One Health surveillance. The findings can therefore be interpreted along a single logical axis: BF11 combines an ST5/CC5, serogroup IIb/1/2b background with phenotypic resistance to multiple clinically relevant antimicrobials, retained virulence-associated loci, stress/persistence-associated determinants, multiple prophage regions, and absence of detected plasmids. cgMLST divergence further indicates that BF11 was not closely matched to genomes currently represented in the queried databases within the applied allelic threshold (i.e., ≤10 allelic differences).

The detected resistance-associated genes can be linked to the observed phenotypic resistance through known antimicrobial resistance mechanisms. Resistance (intermediate resistance) based on the MIC results of Andritsos et al. [37] to fluoroquinolones (e.g., CIP) may be associated with mutations or intrinsic features of DNA gyrase subunits (*gyrA*, *gyrB*), as well as efflux-related mechanisms such as *norB*. Reduced susceptibility to beta-lactams (e.g., P, MEM) may be related to alterations in cell wall synthesis pathways involving genes such as *murA*, *ddl,* and regulatory systems such as *liaFSR*, which contribute to cell envelope stress responses. Resistance to tetracyclines may be partially explained by the presence of *vga(G)*, which is associated with antimicrobial target protection mechanisms. SXT resistance may be linked to genes involved in folate biosynthesis pathways (e.g., *folA*, *folP*, *dfr*), affecting the antimicrobial target pathway. Overall, the resistance phenotype observed in strain BF11 likely reflects the combined effect of intrinsic resistance determinants, regulatory systems, and efflux-associated mechanisms rather than the presence of a single dominant acquired resistance gene.

MLST classified *L. monocytogenes* BF11 as an ST5 (CC5) strain (Table 4), a sequence type repeatedly associated with persistence in food processing environments. ST5 strains are frequently implicated in long-term colonization of production facilities and recurrent contamination events, reflecting their capacity to tolerate environmental stressors and routine sanitation procedures [30,68]. Consistent with this persistence-associated profile, strain BF11 harbored the *bcrABC* cassette and SSI-1, genetic elements known to confer tolerance to quaternary ammonium compounds, acidic conditions, and high salt concentrations [6,22,25]. The co-occurrence of disinfectant tolerance and antimicrobial resistance supports the concept of biocide–antimicrobial co-selection, whereby exposure to sanitizers may indirectly favor the maintenance of antimicrobial resistance [10,25,69].

The virulence-associated gene profile of BF11 indicates that the isolate retains the principal genomic components associated with the canonical pathogenicity repertoire of *L. monocytogenes*. BF11 carried a complete LIPI-1, including *prfA*, *hly*, *plcA*, *plcB*, and *actA*. These genes are central to the intracellular infection cycle of *L. monocytogenes*: *prfA* regulates major virulence determinants, *hly* encodes listeriolysin O, *plcA* and *plcB* encode phospholipases involved in vacuolar escape, and *actA* is associated with actin-based intracellular motility and cell-to-cell spread [2,18,19,20,21]. BF11 also carried several internalin genes, including *inlA*, *inlB*, *inlC*, *inlF*, *inlK*, and *inlP*. Among these, *inlA* and *inlB* are particularly relevant because they are major invasion-associated determinants of *L. monocytogenes* [2,19,20,21]. Sequence analysis showed that *inlA* was full-length and lacked premature stop codons. This finding is relevant because premature stop codons in *inlA* have been associated with truncated Internalin A and reduced invasion of human intestinal epithelial cells, and are reported more frequently among food isolates than among human listeriosis isolates [70,71]. The presence of an intact *prfA* sequence further supports retention of the regulatory component required for coordinated expression of major virulence genes. In addition, sequence variation was detected in *inlB* relative to reference genomes, suggesting the presence of a distinct allelic variant. Overall, these findings support the presence of a preserved virulence-associated genomic repertoire in BF11.

Phylogenomic analyses revealed that, although strain BF11 retains an ST5 genomic backbone, it clusters in close proximity to well-characterized Lineage I outbreak-associated strains such as F2365 and Scott A (Figure 3, Figure 4 and Figure 5). This apparent phylogenetic incongruence, supported by ANI and proteome-based analyses, indicates a mosaic genome architecture shaped by recombination and horizontal gene transfer. Recombination-driven convergence between persistence-associated ST5 backgrounds and virulence-associated Lineage I traits has been documented previously and represents a key mechanism in the adaptive evolution of *L. monocytogenes* [10,70,72]. In *L. monocytogenes* BF11, this convergence provides a mechanistic explanation for the coexistence of environmental resilience and virulence-associated characteristics.

Analysis of the mobilome further highlighted the potential contribution of phage-mediated genome remodeling to the BF11 genome. Four integrated prophage regions were detected, assigned to *Listeria* phages phi A006, phi LP-101, vB_LmoS_188, and B025. In *L. monocytogenes*, prophages are important components of the accessory genome and can contribute to strain diversification through integration, excision, genome rearrangement, and gene gain or loss. Horizontal gene transfer in this species has been associated with plasmids, conjugative transposons, recombination events, and prophages, indicating that mobile genetic elements can introduce new genetic information into otherwise highly conserved genomic backgrounds [10]. This is relevant for BF11 because the isolate combines an ST5/CC5 backbone with multiple accessory genome features, including prophages, recombination-associated genes, and a putative CRISPR-Cas locus. Previous genome-based studies have also emphasized that *L. monocytogenes* populations show heterogeneity in virulence and stress-resistance features despite a relatively conserved core-genome structure, supporting the importance of accessory genome variation in strain differentiation [73,74]. Prophage-associated loci have also been proposed as markers of niche-specific adaptation and persistence in food-processing environments, particularly through *comK*-prophage junctions [74,75]. Therefore, the prophage content of BF11 supports the interpretation that this isolate has undergone accessory-genome remodeling that may contribute to its genomic distinctiveness.

Directly comparable ST5/CC5 MDR pet-food isolates with equivalent WGS-based resistance, virulence, and mobilome characterization remain limited. Therefore, BF11 was compared with available ST5/CC5 strains from food-processing environments and with acquired MDR strains from other clonal backgrounds. ST5 has been detected among food-associated *L. monocytogenes* isolates, while multiresistance patterns were reported in 27.4% of isolates, mainly involving oxacillin, clindamycin, and ceftriaxone; however, all tested isolates were susceptible to P and SXT [76]. ST5/CC5 has also been associated with food-processing environments, including cheese-processing and ready-to-eat meat-processing settings, where ST5/CC5 strains have been linked with persistence-associated features such as SSI-1, *bcrABC*, prophages, and plasmid carriage [77,78].

Comparison with acquired MDR *L. monocytogenes* further emphasizes this distinction. Recent surveillance identified acquired MDR in only 34 of 8344 food isolates from China, mostly belonging to ST9/CC9 rather than ST5/CC5, with MDR frequently associated with plasmid-borne genes such as *dfrG*, *erm*(*B*), *lnu*(*B*), *lsa*(*E*), *tet*(*S*), and *catA* [14]. This is consistent with earlier WGS-based screening of 2862 food isolates from China, in which resistant isolates were uncommon, and with reports of foodborne MDR *L. monocytogenes* carrying plasmid-associated resistance genes such as *cat*, *erm*(*B*), and *tet*(*S*) [79,80]. By contrast, BF11 belonged to ST5/CC5, showed resistance to P, MEM, SXT, TE, and CIP, retained complete LIPI-1 and full-length *inlA*, and had no detected plasmids. Thus, BF11 should be described as a concerning ST5/CC5 MDR pet-food isolate with a notable combination of resistance, virulence-associated, stress/persistence-associated, and mobilome features.

It is worth mentioning that this study has certain limitations. BF11 showed β-hemolysis and a positive CAMP reaction during species confirmation, but no virulence-specific assays were performed. Therefore, the presence of LIPI-1, *prfA*, *hly*, *plcA*, *plcB*, *actA*, and internalin genes should be interpreted as evidence of retained virulence-associated genomic potential. In particular, epithelial-cell invasion, intracellular survival, plaque formation, actin-based motility, macrophage survival, and animal-model virulence were not assessed. Similarly, although BF11 carried stress- and persistence-associated loci such as SSI-1 and *bcrABC*, biofilm formation, sanitizer tolerance, acid or salt tolerance, and other environmental-adaptability phenotypes were not experimentally evaluated. This distinction is important because biofilm formation and environmental persistence in *L. monocytogenes* are condition-dependent and can vary among strains, growth temperatures, nutrient conditions, salt concentrations, and genetic backgrounds [22,23,24,25,30]. Therefore, future studies should combine WGS with phenotypic assays to determine whether the genomic profile observed in BF11 translates into increased host–cell invasion, intracellular survival, biofilm formation, sanitizer tolerance, or persistence under food/feed-processing conditions.

From a One Health perspective, the isolation of *L. monocytogenes* BF11 strain from pet food is particularly significant because this matrix may connect food/feed production, animals, handlers, and household environments. Raw and minimally processed pet foods are increasingly recognized as potential reservoirs of zoonotic and antimicrobial-resistant bacteria, yet they remain comparatively under-surveilled relative to foods intended for human consumption [35,36]. *L. monocytogenes* has been detected in commercial frozen raw meat-based diets for dogs and cats, and broader pet-food surveys have recovered *Listeria*, including *L. monocytogenes*, and *Salmonella* from raw pet-food products [35,81]. Potential exposure routes include handling of contaminated raw pet food, contact with feeding bowls, utensils, storage containers, kitchen surfaces, direct animal contact, and possible fecal shedding after ingestion [35,36]. These routes are especially relevant for vulnerable household members, veterinary personnel, animal handlers, and people caring for service or working dogs. The genomic profile of strain BF11 illustrates how selective pressures in food and feed production environments can drive the emergence of *L. monocytogenes* strains that unite multidrug resistance, environmental persistence, and virulence-associated traits, underscoring the need for integrated genomic surveillance and targeted control strategies across food, feed, and veterinary sectors.

## 5. Conclusions

This study identifies *Listeria monocytogenes* strain BF11 as an MDR strain from raw pet food with a concerning genomic and phenotypic profile. BF11 belonged to serogroup IIb/serovar 1/2b and ST5/CC5, and exhibited resistance to multiple clinically relevant antimicrobials. Whole-genome analysis identified resistance-associated loci, stress/persistence-associated determinants, multiple prophage regions, putative CRISPR-Cas elements, and no detected plasmids under the analytical conditions used.

BF11 also retained major virulence-associated genomic components, including complete LIPI-1, intact *inlA*, and *prfA*, indicating retained virulence-associated genomic potential and absence of a common *inlA*-mediated attenuation marker. Similarly, the detection of SSI-1 and *bcrABC* supports possible stress- and persistence-associated potential.

The isolation of an MDR *L. monocytogenes* ST5/CC5 strain from raw pet food is relevant within a One Health framework because this matrix may connect food/feed production, animals, handlers, and household environments. The combination of multidrug resistance, disinfectant tolerance, and virulence-associated traits in the BF11 strain represents a particularly concerning profile, especially given its isolation from pet food. These findings highlight *L. monocytogenes* strain BF11 as an example of how selective pressures in food and feed production systems can drive the emergence of *L. monocytogenes* strains that unite persistence and pathogenicity. Enhanced genomic surveillance, improved sanitation strategies, and prudent antimicrobial use across food, feed, and veterinary sectors are therefore essential to limit the dissemination of strains with similar high-risk characteristics.

## Figures and Tables

**Table 1:** Quality assessment of the assembled genome for *L. monocytogenes* BF11.

Strain	Completeness (%)	Contamination (%)	Heterogeneity (%)	Number of Contigs (≥300 bp)	N50 (bp) ^1^	Genome Size (bp)
BF11	99.45	0	0	1	2,993,949	2,993,949

^1^ The sum of the lengths of all contigs of size N50 or longer contain at least 50% of the total genome sequence.

**Table 2:** Advanced quality assessment of the assembled genome for *L. monocytogenes* BF11.

Strain	Kraken 2 Contaminants (%)	Typing Loci Detected (%)	Total Assembly Length Deviation (%) ^1^	Percentage of Complete BUSCO Genes (%) ^2^	ConEst 16S	N’s per 100 kbp
BF11	0	99.89	1.68	99.20	Not contaminated	0

^1^ Percent deviation from the expected genome size; ^2^ Benchmarking Universal Single-Copy Orthologs which is used to estimate the completeness of an assembly.

**Table 3:** Properties of the assembled genome for *L. monocytogenes* BF11.

Strain	CDS ^1^	GC Content (%)	Repeat Regions	rRNA	tRNA
BF11	2948	37.98	20	18	68

^1^ CDS (protein-coding sequences).

**Table 4:** MLST and pathogenicity of the *L. monocytogenes* strain BF11 isolated from pet food.

Strain	rMLST Taxonomic Identification	Serogroup, Serovar	Lineage	MLST	CC	Human Pathogen ^1^
BF11	*Listeria monocytogenes*—16013 (rST)	IIb, 1/2b	I	ST5	CC5	Yes (90.9%)

^1^ Whether the microorganism is predicted as human pathogenic (yes or no) and the probability of being human pathogenic (inside the parenthesis).

**Table 5:** Antimicrobial resistance genes of the *L. monocytogenes* BF11 isolate.

Antimicrobial Mechanism	Genes
Antimicrobial inactivation enzyme	*fosX*
Antimicrobial target in susceptible species	*alr*, *ddl*, *dxr*, *TUFM, GFM1*, *folA*, *dfr*, *folP*, *gyrA*, *gyrB*, *inhA*, *fabI*, *iso-tRNA*, *kasA*, *murA*, *rho*, *rpoB*, *rpoC*, *s10p*, *s12p*, *gltA*, *gltB*, *tagB*, *vga*(*G*), *norB*, *lmo0919*, *sul*, *lmo1695*
Antimicrobial target modifying enzyme	*rlmA(II)*
Antimicrobial target replacement protein	*fabK*, *fabL*
Gene conferring resistance via absence	*gidB*
Protein altering cell wall charge conferring antimicrobial resistance	*gdpD*, *mprF*, *pgsA*
Regulator modulating expression of antimicrobial resistance genes	*liaF*, *liaR*, *liaS*
Environmental stress	*bcrABC*, *Stress Survival Islet 1* (*SSI-1*)

## Data Availability

The original data regarding the microbial strain used in the study are included in the article and supplementary material of Andritsos et al. (2025) [37], while they are publicly available at https://doi.org/10.3390/vetsci12090844 and https://www.mdpi.com/article/10.3390/vetsci12090844/s1 (accessed on 16 February 2026). Genome accession number for *L. monocytogenes* strain BF11 is BioProject ID: PRJNA1464443 (BioSample accession: SAMN59708363).

## Supplementary Materials

The following supporting information can be downloaded at: https://www.mdpi.com/article/10.3390/microorganisms14051097/s1, Table S1: Reference genomes of *Listeria* spp. strains used in this study for phylogenomic and comparative analyses.
