# Supplementary material for: Whole-Genome Sequencing and Genomic Characterization of a Multi-Drug Resistant Phenotype of Listeria monocytogenes Isolated from Pet Food
Source: Microorganisms. 2026 May 12;14(5):1097. doi: 10.3390/microorganisms14051097 (PMC13209999; doi:10.3390/microorganisms14051097)
Supplement: Supplementary file 1 [file microorganisms-14-01097-s001.zip › microorganisms-4271931-supplementary.pdf]

microorganisms

Whole-Genome Sequencing and Genomic Characterization of a Multi-Drug Resistant Phenotype of *Listeria monocytogenes* Isolated from Pet Food

Antonia Mataragka [1,2], Marios Mataragas [3,*], Nikolaos Tzimotoudis [4], Ioannis Galiatsatos [5], Panagiota Stathopoulou [5], Spiros Paramithiotis [6], John Ikonomopoulos [2] and Nikolaos D. Andritsos [1,*]

1 Department of Food Science and Technology, School of Agricultural Sciences, University of Patras, 2 G. Seferi Str., GR-30100 Agrinio, Greece; antonia.mataragka@gmail.com
2 Laboratory of Anatomy and Physiology of Farm Animals, Department of Animal Science, School of Animal Biosciences, Agricultural University of Athens, 75 Iera Odos Str., GR-11855 Athens, Greece; ikonomop@aua.gr
3 Department of Dairy Research, Institute of Technology of Agricultural Products, Hellenic Agricultural Organization "DIMITRA", 3 Ethnikis Antistaseos Str., Katsikas, GR-45221 Ioannina, Greece
4 Hellenic Army Biological Research Centre, 6 Taxiarchou Velliou Str., P. Penteli, GR-15236 Attica, Greece; n.p.tzimotoudis@army.gr
5 Laboratory of Systems Microbiology and Applied Genomics, Department of Sustainable Agriculture, School of Agricultural Sciences, University of Patras, 2 G. Seferi Str., GR-30100 Agrinio, Greece; jgalia96@gmail.com (I.G.); panstath@upatras.gr (P.S.)
6 Department of Biological Applications and Technology, School of Health Sciences, University of Ioannina, Ioannina Campus, GR-45110 Ioannina, Greece; paramithiotis@uoi.gr
* Correspondence: mmatster@elgo.gr (M.M.); nandritsos@upatras.gr (N.D.A.); Tel.: +30-26510-94780 (M.M.); +30-26410-74176 (N.D.A.)

Supplementary Materials

Table S1. Reference genomes of *Listeria* spp. strains used in this study for phylogenomic and comparative analyses.

Strain	Accession Number	Source	Notes
Listeria monocytogenes BF11	BioProject ID: PRJNA1464443 (BioSample accession: SAMN59708363)	Pet Food	NCBI
Listeria monocytogenes F2365		Clinical (reference)	NCBI, Serotype 4b, clinical outbreak strain
Listeria monocytogenes J1816	NC_002973.6	Clinical	NCBI
Listeria monocytogenes CC121 CT14745	NZ_CP006612.1	Human	NCBI
Listeria monocytogenes CC121	Cluster representative	Food	NCBI
Listeria monocytogenes CC121 CT1011	Cluster representative	Human	NCBI
Listeria monocytogenes CC121	Cluster representative	Food Pie	NCBI
Listeria monocytogenes CC121 CT909	Cluster representative	Human	NCBI
Listeria monocytogenes CC101 CT14750	Cluster representative	Human	NCBI
Listeria monocytogenes FSL F6-684	Cluster representative	Food / Environment	NCBI
Listeria monocytogenes NCTC10357	NZ_CP009242.1	Reference	NCBI
Listeria monocytogenes 10403S	LS483390.1	Laboratory strain	NCBI
Listeria monocytogenes EGD-e	NC_017544.1	Reference	NCBI
Listeria monocytogenes FSL J1-208	NC_003210.1	Food / Environment	NCBI
Listeria innocua CLIP11262	NZ_CP009243.1	Environmental	NCBI, used as outgroup
Listeria monocytogenes F2365	NC_003212.1	Clinical	BV-BRC
Listeria monocytogenes Scott A	265669.9 / 1639.8539	Clinical	BV-BRC
Listeria monocytogenes EGD-e	1027396.3 / 1639.7647 / 1639.8620	Reference	BV-BRC
Listeria monocytogenes R479a	169963.234 / 1639.15813	-	BV-BRC
Listeria monocytogenes 08-5578	1437838.3	ST5 reference	BV-BRC
Listeria monocytogenes LM10	653938.3<br>1639.13684	-	BV-BRC
